# ﻿An overview of the Leucospidae (Hymenoptera, Chalcidoidea) of the Arabian Peninsula with description of a new species

**DOI:** 10.3897/zookeys.1189.113635

**Published:** 2024-01-16

**Authors:** Syed Kamran Ahmad, Syeda Uzma Usman, Farmanur Rahman Khan, Hossein Lotfalizadeh, Hassan A. Dawah, Parvez Qamar Rizvi, Prince Tarique Anwar

**Affiliations:** 1 Department of Plant Protection, Faculty of Agricultural Sciences, Aligarh Muslim University, Aligarh – 202002, India Aligarh Muslim University Aligarh India; 2 Department of Zoology, Mohammad Ali Jauhar University, Rampur – 244901, Uttar Pradesh, India Mohammad Ali Jauhar University Rampur India; 3 Department of Biology, Deanship of Educational Services, Qassim University, Buraidah – 51452, Al-Qassim, Saudi Arabia Qassim University Buraidah Saudi Arabia; 4 Department of Plant Protection, East-Azarbaijan Agricultural and Natural Resources Research Center, Agricultural Research, Education and Extension Organization (AREEO), Tabriz, Iran East-Azarbaijan Agricultural and Natural Resources Research Center, Education and Extension Organization (AREEO) Tabriz Iran; 5 Centre for Environmental Research and Studies, Jazan University, P.O. Box 2095, Jazan, Saudi Arabia Jazan University Jazan Saudi Arabia; 6 Department of Zoology, School of Sciences, Aligarh College of Education, Chherat, Aligarh – 202122, India School of Sciences, Aligarh College of Education Aligarh India

**Keywords:** Biodiversity, ectoparasitoids, new species, taxonomy

## Abstract

An overview of the family Leucospidae (Hymenoptera, Chalcidoidea) is provided for the leucospid fauna of the Arabian Peninsula. Two genera containing four species are identified based on morphometrics and colour patterns. One species, *Leucospisayezae* Usman, Anwar & Ahmad, **sp. nov.**, is described. *Leucospiselegans* Klug had been previously recorded from Arabia Felix (= Yemen) and is recorded here for the first time from Saudi Arabia. The status of Leucospisaff.namibica from Yemen has been clarified, and this species is placed here in the genus *Micrapion* Kriechbaumer as *M.clavaforme* Steffan. An updated key and a map showing the distribution of the family Leucospidae in the Arabian Peninsula is provided. The occurrence and color morphs of all leucospid species that have been recorded so far from the region are briefly discussed.

## ﻿Introduction

Members of the family Leucospidae (Hymenoptera, Chalcidoidea) are large chalcid wasps (6–15 mm) and develop as ectoparasitoids on aculeate wasps or bees (Lima and Dias 2018). They are mostly dark brown, red, or yellow, with a patterned, orange or white body, metafemur enlarged with teeth, and strongly curved metatibia. Females typically have a recurved ovipositor which lies along the dorsal side of the metasoma. Leucospids are cosmopolitan in their distribution but rarely encountered, and there are 144 described species worldwide which belong to four genera (Noyes 2019).

The family is mostly represented by the genus *Leucospis* Fabricius, which accounts for more than 86% of the total number of species. Bouček (1974) provided a comprehensive taxonomic revision of the Leucospidae and provided separate keys to American, African, and Asiatic-Australian *Leucospis* species. More recently, Ye et al. (2017) recognized and provided a key to 12 *Leucospis* species from China. In the Arabian Peninsula six valid leucospid species have been reported so far: *Leucospiselegans* Klug (Bouček 1974 from Saudi Arabia [= Arabia Felix i.e., Yemen]; Schmid-Egger 2010 from UAE), *L.insularis* Kirby (Kirby 1900 from Yemen), *L.vanharteni* Schmid-Egger (Schmid-Egger 2010 from UAE), *L.arabica* Gadallah & Soliman, *L.africana* Cameron, and *Micrapionclavaforme* Steffan (Gadallah et al. 2018 from Saudi Arabia).

Schmid-Egger (2010) tentatively identified two specimens of *Leucospis* as L.aff.namibica. On close examination of his figure (Schmid-Egger 2010: 321, pl. 3), there is no doubt that the specimens are not a *Leucospis* but *Micrapionclavaforme* Steffan instead. Some other important works on the Leucospidae from the Middle East were provided by Hesami et al. (2005), Lotfalizadeh and Fakhrzadeh (2012), Madl and Schwarz (2014), and Kareem et al. (2020). Schmid-Egger (2010) provided a key to four species of from the UAE and Yemen, and Gadallah et al. (2018) keyed five species of Leucospidae (one *Micrapion* Kriechbaumer species and four *Leucospis* species) from Saudi Arabia.

**Figure 1.**
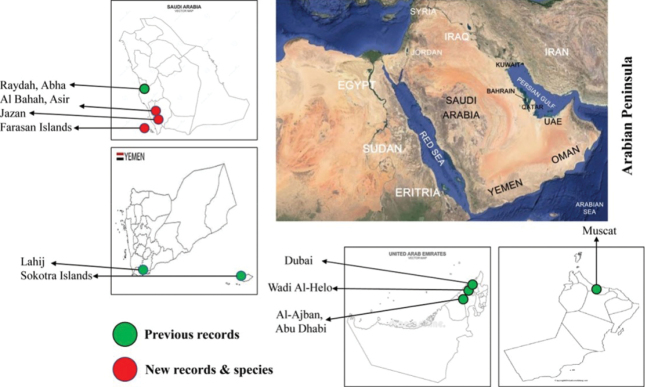
Distribution map of leucospid species in the Arabian Peninsula.

Here we describe a new species of *Leucospis* from Jazan, Saudi Arabia, and also report on some of the known *Leucospis* and *Micrapion* species. Diagnoses and illustrations of types are provided for two of them, *L.insularis* and *L.africana*. An extended and modified version of map (Fig. 1) and the key given by Schmid-Egger (2010) and Gadallah et al. (2018) is also provided to place our newly described species. All species treated herein are fully illustrated.

## ﻿Methods

The study is based on the materials collected from three provinces of Saudi Arabia, Asir, Najran, and Jazan (Table 1). The specimens were collected mainly by one of two methods, either by sweep net (SN) or in a Malaise trap (MT). The collected specimens were primarily stored in 80% ethanol and were later mounted on rectangular cards. For each species, one pair of wings were removed and mounted on a slide. For the new species, *Leucospisayezae*, the head and a hind leg was removed and mounted on the same card while one antenna was mounted on slide by following the methods described by Noyes (1982) with modifications as mentioned by Anwar et al. (2020). Photographs of card-mounted specimens were taken using a Nikon SMZ 1000 stereozoom binocular microscope. Figs 3B, 5B, 8C, D were taken using a video camera and Synaptics Automontage software to produce a montage image of the species. Photographs of the slide-mounted parts were taken with a Leica DFC295 digital camera attached to a Leica DM 2500 compound microscope with automountage facility. The final figures were prepared using Adobe Photoshop v. 7.0.

**Table 1.** List of sampling sites with coordinates, altitude, and sampling methods for Leucospidae collected from Southwest of Saudi Arabia.

Locality	Coordinates	Altitude (m)	Method
Abha, Hay Al-Menhel, vegetable farm	18°12'N, 42°29'E	2214	MT
Abha, Hay Al-Nusub (Abha Farm Centre) vegetable farm	18°13'N, 42°30'E	2226	MT, SN
Jazan, Farasan Island, Aziz Yousef Village	16°40'N, 42°50'E	3	MT, SN
Najran, Al-Shurfa	17°31'N, 44°15'E	1342	MT

Measurements were made with the use of an ocular micrometer attached to the eyepiece of the microscope and were later converted into micrometers (µm). All the determined and type materials were deposited at the Insect Collections Department of Zoology, Aligarh Muslim University, Aligarh, Uttar Pradesh, India.

The terms mentioned in the text follow Bouček (1974) and Lima and Dias (2018).

List of abbreviations used in the text: **AOL**, anterior-ocular length; **MOD**, midian ocellar diameter; **OCL**, ocular–occipit length; **OOL**, ocello–ocular line; **POL**, posterior ocellar line; **psa**, parascrobal area; **F**, antennal ﬂagellomere; **PMV**, postmarginal vein of fore wing; **STV**, stigmal vein; **GT**, gastral or metasomal tergite; **MT**, Malaise trap; **SN**, Sweep net.

The following acronym is used for the depository:

**BMNH** Natural History Museum [formerly British Museum (Natural History)], Department of Entomology, London, UK;

**KSMA**King Saud University Museum of Arthropods, Plant Protection Department, College of Food and Agriculture Sciences, King Saud University, Riyadh, Saudi Arabia;

**MNHN**Muséum National d’Histoire Naturelle, Paris, France;

**NMWC**The National Museum of Wales, Cardiff, UK;

**ZMHU**Zoological Museum, Humboldt University, Berlin, Germany;

**ZDAMU**Department of Zoology, Aligarh Muslim University, Aligarh, India.

## ﻿Results

Four species, including one new species, are among the materials examined. These belong to one of two genera, either *Leucospis* or *Micrapion*. The family Leucopsidae has seven species in total known from the Arabian Peninsula, and all seven species are keyed below.

### ﻿An updated key to females of Leucospidae from the Arabian Peninsula

Modified from Schmid-Egger 2010 and Gadallah et al. 2018.

**Table:** 

1	Clypeus curved convexly at posterior margin and without a median tooth; mandibles thin, setose, and notched at apex; gaster distinctly clavate, basally narrow (Fig. 9A, C); GT4 with hind margin always produced backward and sharply angulate (Fig. 9A, C); GT6 fused to epipygium (gaster with coarse punctures; GT1 without band and with shiny interspaces; apical band of GT5 0.5× as broad as length of sheaths; metafemur relatively slender)	***Micrapionclavaforme* Steffan**
–	Clypeus bilobed at posterior margin and often with a median tooth; mandibles robust, without setae; lower tooth stronger; gaster less clavate (Figs 3C, 4A, 5B, 7B, 8B); GT4 posteriorly straight; GT6 distinctly separated from epipygium	**2 (genus *Leucospis* Fabricius)**
2	Pronotum with three distinct transverse carinae (Figs 3A, 5A, 7A, 8A)	**3**
–	Pronotum with at most two transverse, less-developed carinae (Fig. 4A)	**6**
3	Ovipositor short, not reaching anterior margin of GT5 (Fig. 7A)	***Leucospisafricana* Cameron**
–	Ovipositor long, reaching at least posterior margin of GT4 or beyond (Figs 3A, 4A, 5B, 8B)	**4**
4	Discal carina on pronotum weak and straight (Fig. 8A); metafemur slender with nine ventral teeth, basal tooth angular and pointed (Fig. 8A); ovipositor distinctly reaching beyond (hind fifth) posterior margin of GT1 (Fig. 8B)	***Leucospisinsularis* Kirby**
–	Discal carina on pronotum strong and angulate; metafemur oval with eight or nine ventral teeth, basal tooth triangular and robust; ovipositor hardly reaching posterior margin of GT1	**5**
5	Metafemur with nine ventral teeth (Fig. 3B); ovipositor hardly reaching posterior margin of GT4 (Fig. 3C)	***Leucospisayezae* Usman, Anwar & Ahmad sp. nov.**
–	Metafemur with eight ventral teeth (Fig. 5A); ovipositor clearly reaching posterior margin of GT1 (Fig. 5B)	***Leucospiselegans* Klug**
6	Pronotum red or orange, except black at base of mesopleuron; middle teeth of metafemur distinctly longer than basal triangular tooth	***Leucospisvanharteni* Schmid-Egger**
–	Pronotum dark brown, except with a transverse yellow strip posteriorly between preapical and marginal carinae, continuing to lateral panel of pronotum as an oblique marking above ventral depression of panel (Fig. 4A); basal tooth of metafemur longer and more robust than any of the following teeth	***Leucospisarabica* Gadallah & Soliman**

### ﻿Taxonomy

#### ﻿New species

##### ﻿ Leucospis ayezae

Taxon classificationAnimaliaHymenopteraLeucospidae

Usman, Anwar & Ahmad sp. nov.

EE2A77D0-0110-5808-B26D-D57F279600D1

https://zoobank.org/02A1622A-412E-423C-B50C-028EE20D31EF

Figs 2 , 3

###### Type material.

***Holotype***: Saudi Arabia • ♀; Asir, Abha, Hay Al-Nusub; 18°13'N, 42°30'E; 2226 m alt.; 24.vii.2013; H.A. Dawah leg.; ZDAMU Reg. No. HYM.CH.873, body, dissected head with one antenna and one dissected hind leg on card; one pair of fore wing and antenna on slide under two coverslips, slide HYM.06.

###### Diagnosis.

The new species is similar to *L.insularis* in having a yellow band on the pronotum and scutellum and distinct discal, preapical, and marginal carinae, but the new species differs from *L.insularis* as follows: discal carina on pronotum strong and angulate (discal carina on pronotum weak and straight in *L.insularis*); metafemora oval with eight ventral teeth, basal tooth triangular and robust (metafemora slender with nine ventral teeth, basal tooth angular and pointed in *L.insularis*); pubescence on sides of propodeum and metatibia relatively short and less dense (pubescence on sides of propodeum and metatibia long and more dense in *L.insularis*) ovipositor hardly reaching posterior margin of GT4 (ovipositor distinctly reaching beyond posterior margin of GT1 in *L.insularis*).

###### Description.

**Figure 2.**
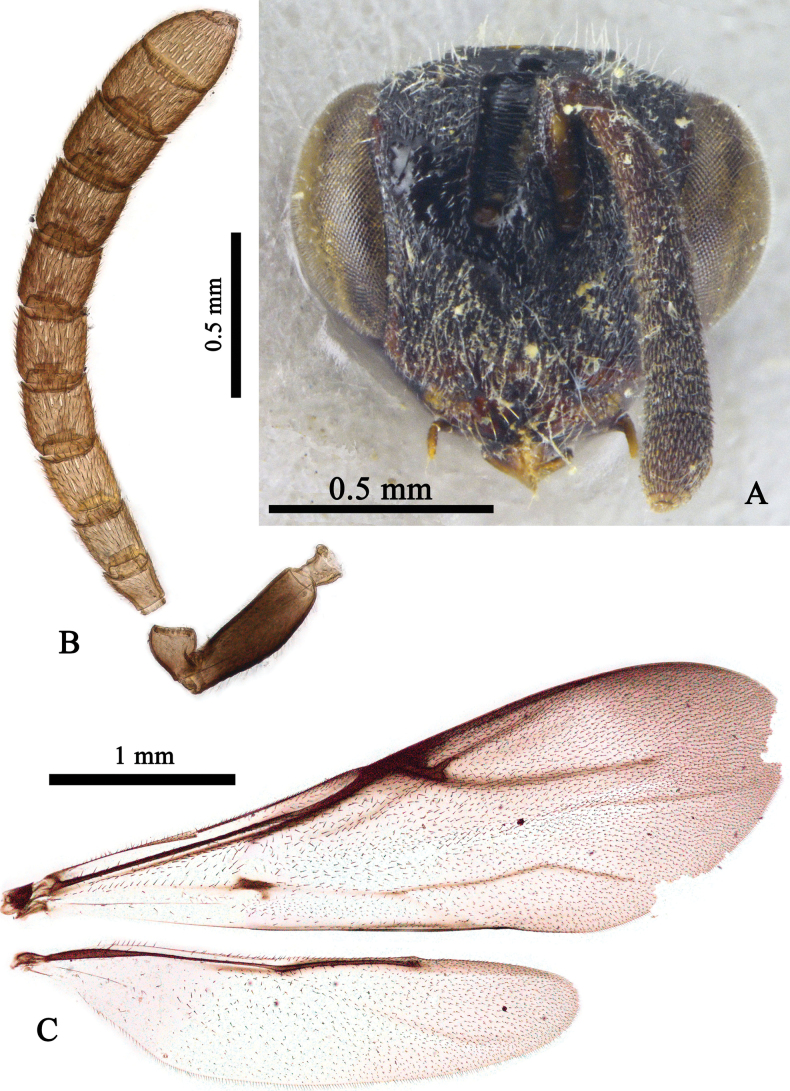
*Leucospisayezae* Usman, Anwar & Ahmad sp. nov. holotype, female **A** head, frontal view **B** antenna **C** wings.

**Figure 3.**
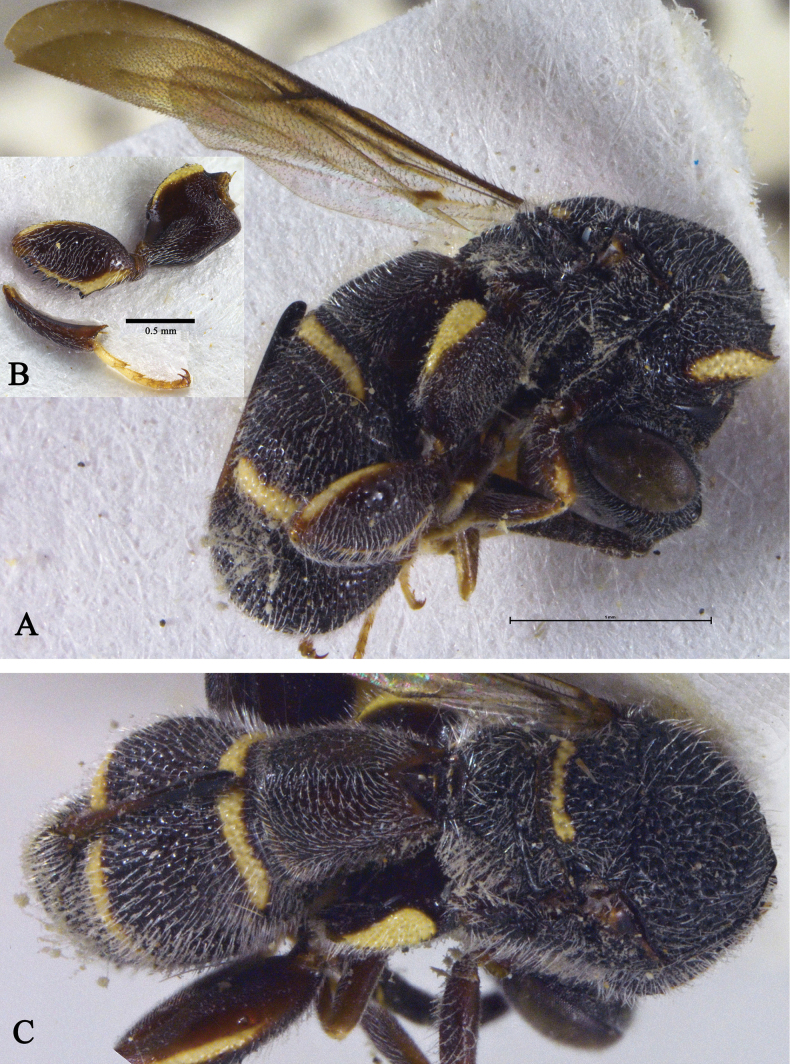
*Leucospisayezae* Usman, Anwar & Ahmad sp. nov. holotype, female, habitus **A** lateral view **B** dorsal view.

***Colour*** (Figs 2A, 3). Head dark brown; maxillary and labial palps yellowish brown; antenna dark brown except scape with posterior margin yellow. Mesosoma dark brown except a yellow transverse strip in front of discal carina, not continuing to sides of pronotum, and a narrow, transverse yellow strip on scutellum just above apex. Gaster largely reddish to dark brown, with transverse yellow strips medially on GT4 and apically on GT5. Basal two-thirds of ovipositor reddish brown; the rest dark brown. Pro- and mesofemur brown, with yellow tips where joining tibia; pro- and mesotibia reddish brown, with their margins yellow; hind legs dark brown except apex of coxa in ventral view and margins of femur yellow; all tarsi yellow. Fore wing below PMV and in apical half strongly infuscate, the rest hyaline.

***Head*** (Fig. 2A). Head in frontal view 1.2× as broad as high and as wide as posterior margin of pronotum; less densely punctuate, medially at psa smooth, setae on face and eyes silvery, erect, and less dense; POL 2× OOL and 3× MOD; scrobe 1.5× as wide as parascrobal area, transversely carinate; occipital carina distinctly visible between and beyond posterior ocelli; malar space 0.35× eye height and as long as F7; flagellum with erect, black setae; F1 widened apically, as long as broad and shortest of all funicular segments individually; F3–F5 subequal in length; F7 longest; clava 1.7× as long as broad, distinctly longer than F7 and F8 combined (Fig. 2B); mandible tridentate.

***Mesosoma*** (Fig. 3A, C). Mesosoma densely punctuate, punctures setigerous, with dense, long, pale setae; setae denser on propodeal callus; discal, preapical, and marginal carinae well developed, raised, and angulate; posterior margin of scutellum with punctures in a line with margins and with carina; propodeum medially with a complete carina. Hind leg with coxa punctuate, carinate and subserrate posterodorsally; metafemur oval, 1.9× as long as broad, punctuate, and setose, with eight ventral teeth; basal tooth robust, 3–5 longer than rest and subequal (Fig. 3B); metatibia with spine subequal to spur. Fore wing 3.2× as long as broad, with dense, black setation towards apical margin; STV with bifurcate uncus; uncus longer than apical process of stigmal vein (Fig. 2C). Hind wing 4.4× as long as broad (Fig. 2C).

***Metasoma*** (Fig. 3A, C). Gaster moderately punctuate, with dense, pale setae; density of setae more at epipygium. GT1 wider than long, interiorly with triangular process attached to petiole and, medially with a raised carina, narrower than GT4 in dorsal view; GT4 with posterior margin entire; ovipositor sheaths long, nearly reaching anterior margin of GT3.

***Measurements*** (holotype, mm): head width:length:height, 1.4:0.7:1.2; AOL, 0.14; MOD, 0.12; OCL, 0.03; OOL, 0.19; POL, 0.38; sh [scrobe height], 0.51; sw [scrobe weight], 0.4; psa, 0.33; ceh [compound eye height], 0.82; mls [malar space], 0.28; antennal segments length:width — radicle, 0.13:0.16; scape, 0.64:0.2; pedicel, 0.24:0.17; F1, 0.16:0.16; F2, 0.22:0.2; F3, 0.25:0.24; F4, 0.25:0.25; F5, 0.25:0.27; F6, 0.25:0.28; F7, 0.28:0.22; F8, 0.24:0.32; clava, 0.56:0.33; pronotum, 0.56; mesoscutum, 0.72; scutellum, 0.61; dorsellum, 0.16; propodeum, 0.24; fore wing length:width, 4.4:1.35; hind wing length:width, 3.1:7; metacoxa, 0.96:0.85; metafemur, 1.24:0.64; metatibia, 1.12; metatarsus, 1.12; petiole, 0.16:0.37; gaster, 2.8; GT1, 0.9; GT3, 0.1; GT4, 0.4; GT5, 0.7; GT6, 0.2; ovipositor, 1.4; hypopygeum, 0.8.

**Male.** Unknown.

###### Host.

Unknown.

###### Distribution.

Saudi Arabia: Asir.

###### Etymology.

The species name after Ayeza Tarique, daughter of the authors SUU and PTA.

#### ﻿Other species

##### ﻿ Leucospis arabica

Taxon classificationAnimaliaHymenopteraLeucospidae

Gadallah & Soliman, 2018

AA81C5C0-2044-50F1-89B1-2E53BEC7706F

Fig. 4

Leucospis arabica Gadallah & Soliman in Gadallah et al. 2018: 2079, female, male. Holotype, female (KSMA), Saudi Arabia (Jazan, Farasan Islands), not examined.

###### Materials examined.

8♀, 9♂. Saudi Arabia • Jazan, Farasan Island, Aziz Yousef Village; 16°40'N, 42°50'E; 3 m alt.; 6♀, 9♂ (each on cards; 2 females, 1 male with one pair of wings on slide under 1 coverslip, slide No. HYM.02, 03, 11), 15.v.2017; S.K. Ahmad leg.; 2♀ (on cards); 13.v.2017; H.A. Dawah leg.; ZDAMU.

**Figure 4.**
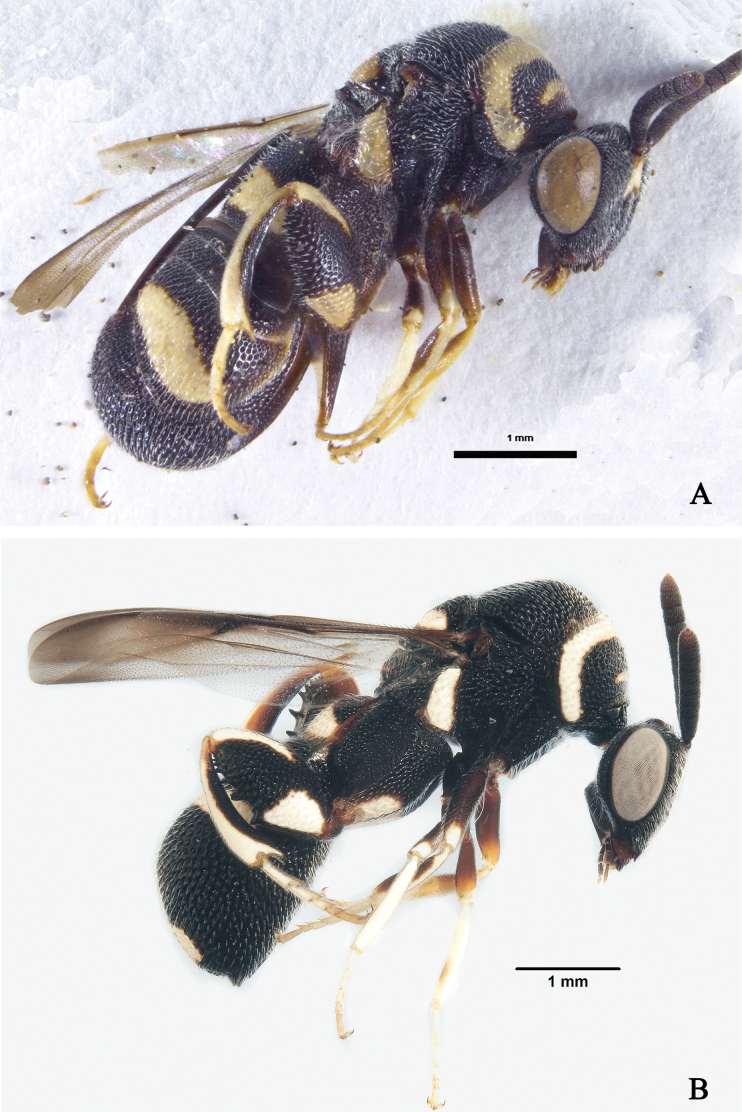
*Leucospisarabica* Gadallah & Soliman, habitus **A** female, lateral view **B** male, lateral view.

###### Remarks.

The examined specimens were collected from the type locality and differ from the holotype in size. The females were 5–15 mm long and males 2–10 mm long. In both sexes, the size of the yellow patch on the metafemur varies minute to broad.

###### Host.

Unknown.

###### Distribution.

Saudi Arabia: Jazan (Farasan Islands) and Egypt (Sinai Peninsula).

##### ﻿ Leucospis elegans

Taxon classificationAnimaliaHymenopteraLeucospidae

Klug, 1834

567FD32A-238B-5453-823D-9D09161FED81

Figs 5 , 6

Leucospis elegans Klug, 1834: 26. Holotype, female (ZMHU), Yemen, not examined.

###### Materials examined.

2♀, 1♂. Saudi Arabia • Jazan, Farasan Island, Aziz Yousef Village; 16°40'N, 42°50'E; 3 m alt.; 2♀ (on cards, one pair of fore wing of one female specimen on slide under 1 coverslip, slide No. HYM.04; one pair of fore wing and antenna of other female specimen on slide under 2 coverslips, slide No. HYM.05), 15.v.2017; S.K. Ahmad leg.; 1♂ (on card, one pair of fore wing and antenna on slide under 2 coverslips, slide No. HYM.10), 1.ii.2015; H.A. Dawah leg.; ZDAMU.

**Figure 5.**
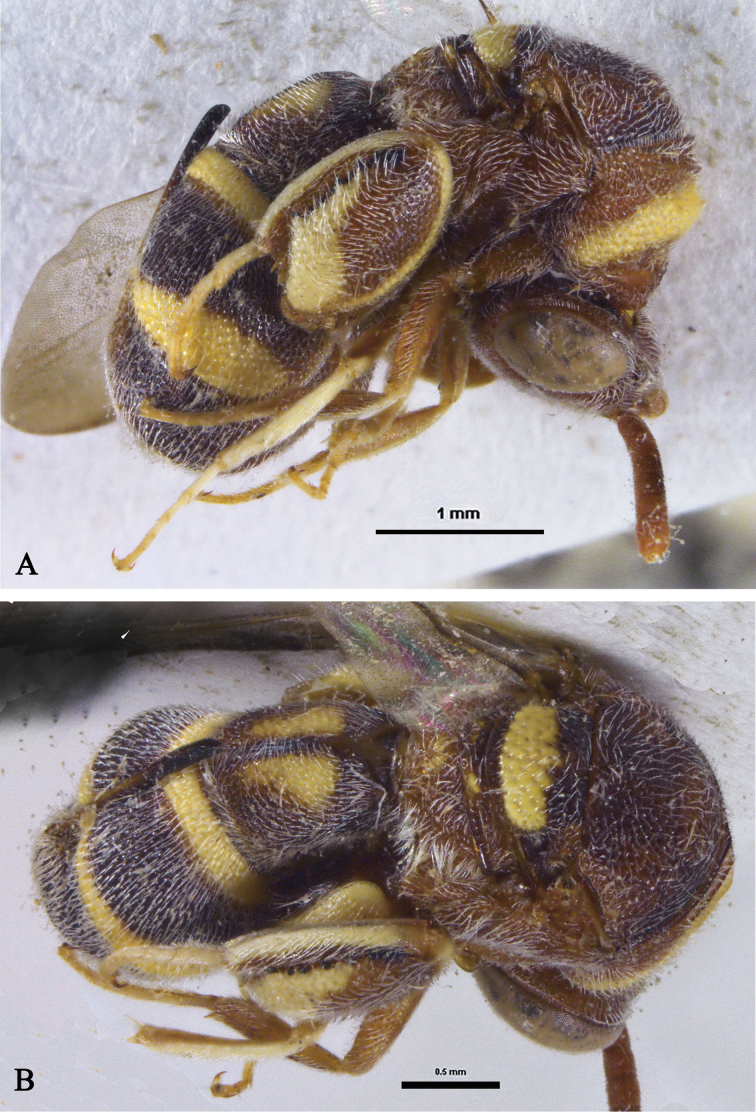
*Leucospiselegans* Klug, female, habitus **A** lateral view **B** dorsal view.

###### Remarks.

This is the first record of *L.elegans* from Saudi Arabia. However, Bouček (1974) included it in the fauna of Saudi Arabia but referred to Arabia Felix, which is a former name for Yemen. He briefly provided a diagnosis of *L.elegans* and described the male for the first time.

**Figure 6.**
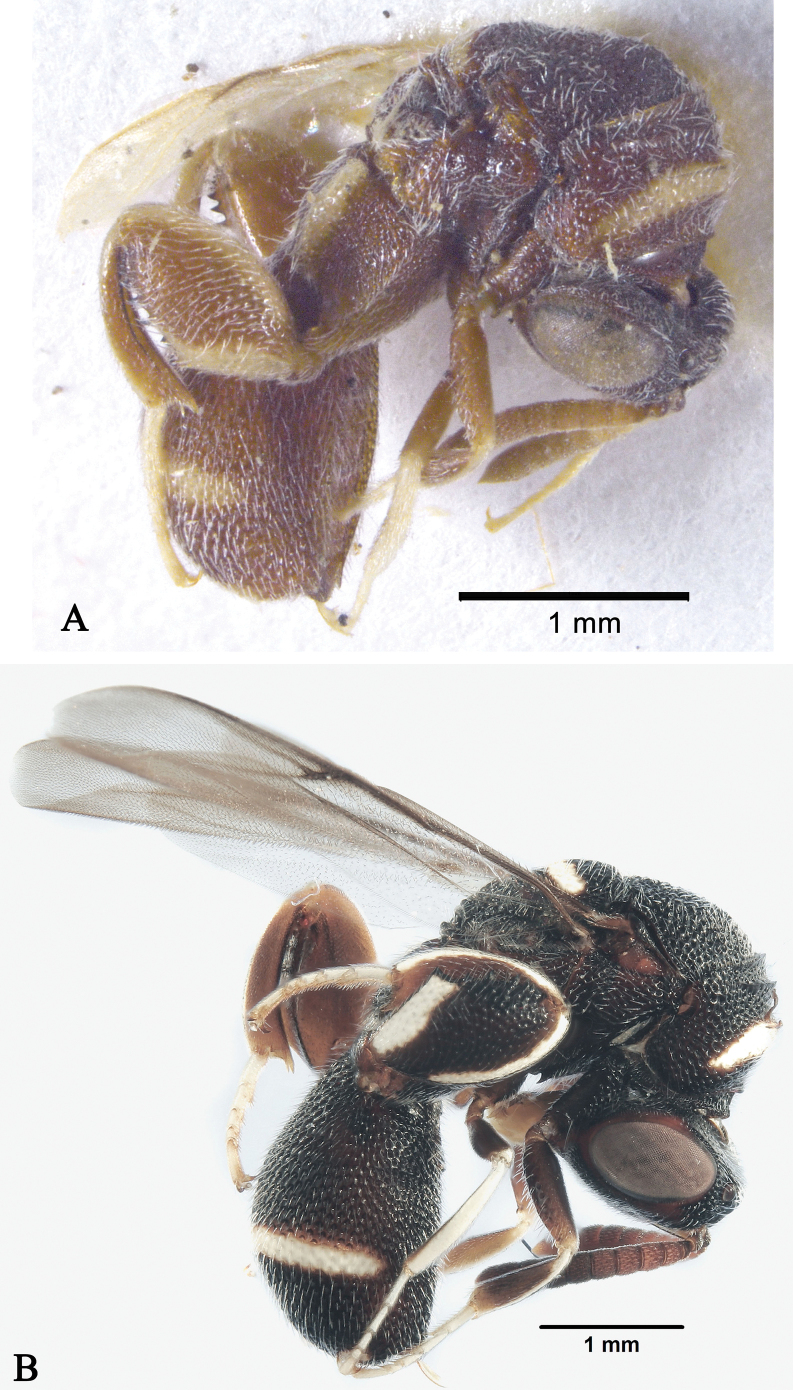
*Leucospiselegans* Klug, males, habitus, lateral view **A** reddish brown-yellow morph **B** dark brown-pale yellow morph.

###### Host.

Unknown.

###### Distribution.

Afrotropical, Palaearctic, Oriental (Klug 1834; Bouček 1959, 1974; Narendran 1986; Schmid-Egger 2010; Madl and Schwarz 2014; Gadallah et al. 2018). Yemen (as Arabia Felix; Bouček 1974). Saudi Arabia (new record).

##### ﻿ Leucospis africana

Taxon classificationAnimaliaHymenopteraLeucospidae

Cameron, 1907

18BC19D1-807B-505E-A605-96F53BAABB12

Fig. 7

Leucospis africana Cameron, 1907: 204. Lectotype, female (BMNH), designated by Bouček 1974: 104, South Africa (Cape Province), examined (illustrations only).

###### Remarks.

Bouček (1974) recorded *L.africana* from several African countries and provided a brief diagnosis of females and described the males. He further recorded its host for the first time. Gadallah at al. (2018) recorded males from Saudi Arabia and provided a detailed diagnosis of males and a key identify it from other species of Saudi Arabia. Here, we figure the lectotype for the first time.

**Figure 7.**
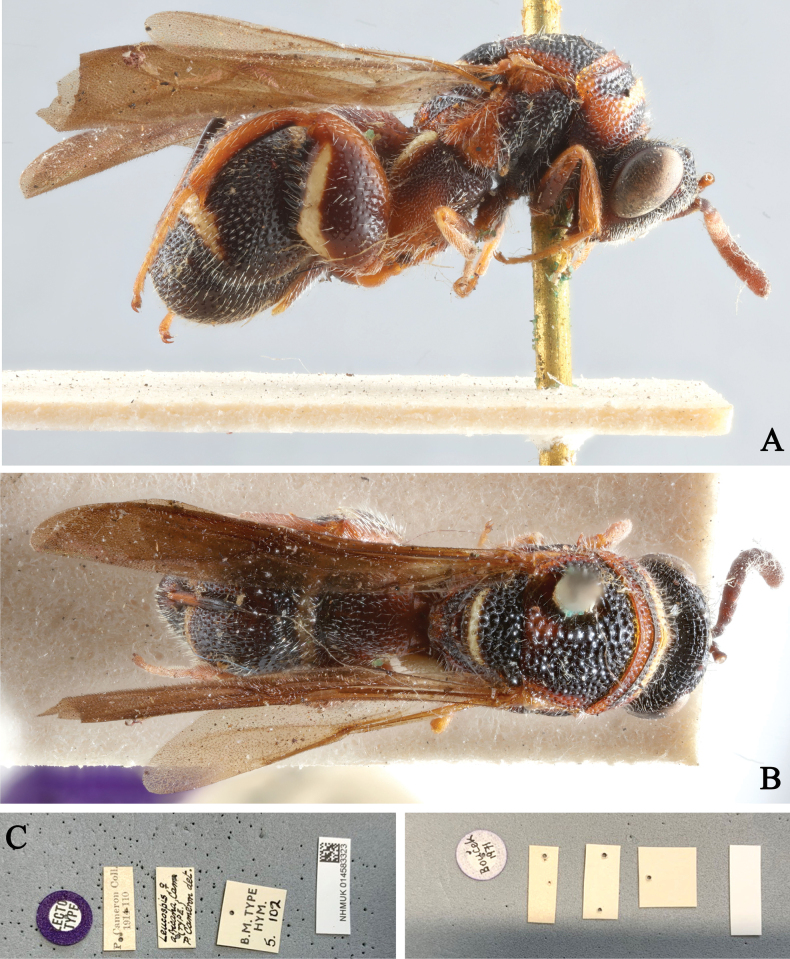
*Leucospisafricana* Cameron, lectotype, female (photographs courtesy of Natalie Dale-Skey Papilloud, BMNH) **A** lateral view **B** dorsal view **C** labels.

###### Host.

*Serapistadenticulata* (Smith) (Hymenoptera, Megachilidae) (Bouček 1974); *Megachilespinarum* Cockerell (Hymenoptera, Megachilidae) (Gess 1981).

###### Distribution.

Afrotropical: Burundi, Central African Republic, Democratic Republic of Congo, Eretria, Ethiopia, Ghana, Ivory Coast, Kenya, Lesotho, Malawi, Mozambique, Nigeria, Rhodesia, South Africa, Tanzania, Uganda, Zambia, Zimbabwe (Cameron 1907; Bouček 1974; Noyes 2019); Saudi Arabia (Gadallah at al. 2018).

##### ﻿ Leucospis insularis

Taxon classificationAnimaliaHymenopteraLeucospidae

Kirby, 1900

951EC5BB-F9A9-546D-88E1-03CD729AA012

Fig. 8

Leucospis insularis Kirby, 1900: 13. Holotype, female (BMNH), Yemen (Socotra Island), examined (illustrations only).

###### Remarks.

*Leucospisinsularis* is only known from the type locality Socotra Islands (Yemen). Bouček (1974) included *L.insularis* in a key to African *Leucospis*. Schmid-Egger (2010) included it his key to Arabian species. Here, we figure the holotype for the first time.

**Figure 8.**
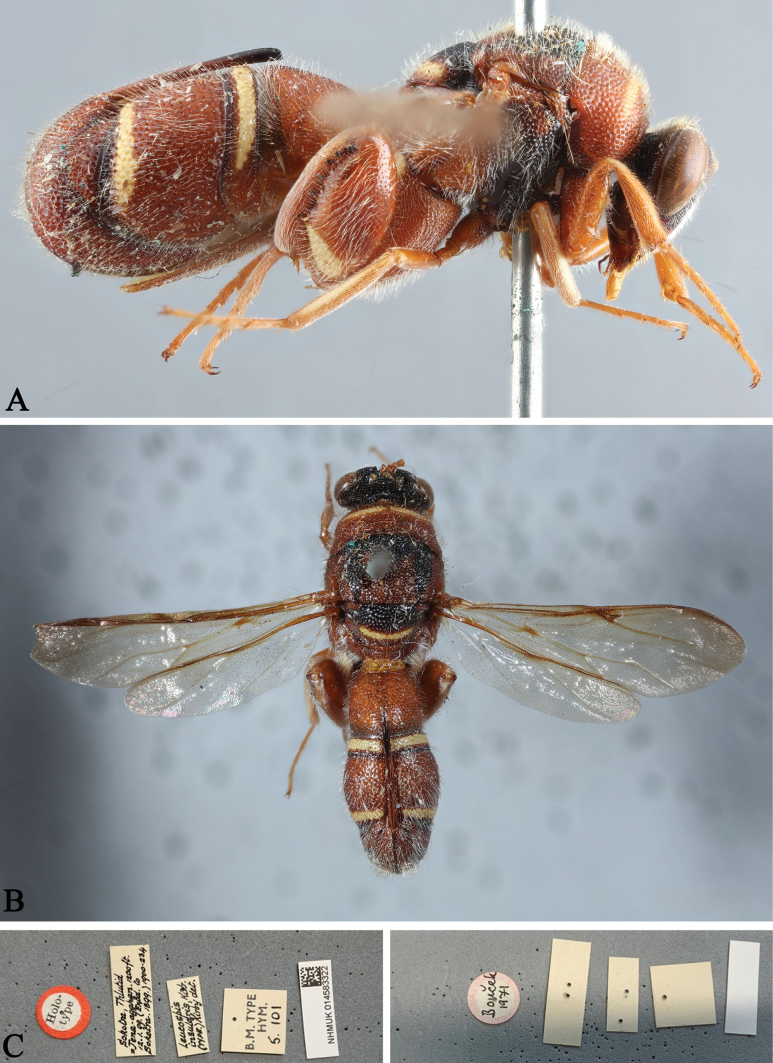
*Leucospisinsularis* Kirby, holotype, female (photographs courtesy of Natalie Dale-Skey Papilloud, BMNH) **A** lateral view **B** dorsal view **C** labels.

###### Host.

Unknown.

###### Distribution.

Afrotropical: Socotra Islands (Yemen) (Kirby 1900).

##### ﻿ Micrapion clavaforme

Taxon classificationAnimaliaHymenopteraLeucospidae

Steffan, 1948

36700FBD-E5B9-5101-BABD-873B3D9A8CEC

Fig. 9

Micrapion clavaforme Steffan, 1948: 85, female. Lectotype, female (MNHN), designated by Bouček 1974: 220, Gabon (Ogowe), not examined.

###### Material examined.

3♀, 2♂. Saudi Arabia • Asir, Abha, Hay Al-Menhel; 18°12'N, 42°29'E; 2214 m alt.; 2♀ (one on card; one on card with one pair of fore wing on slide under 1 coverslip, slide No. HYM.01), 20.xii.2014; H.A. Dawah leg.; Najran • Al-Shurfa, Saleh Maqbol Farm, 17°31'N, 44°15'E; 1342 m alt.; 1♀ (on card, one pair of fore wing on slide under 1 coverslip, slide No. HYM.09), 17.ix.2014; H.A. Dawah leg.; Asir, Abha, Hay Al-Nusub, 18°13'N, 42°30'E; 2226 m alt.; 2♂ (one on card; one on card with one pair of fore wing on slide under 1 coverslip, slide No. HYM.07), 3.vi.2015; H.A. Dawah leg.; ZDAMU.

**Figure 9.**
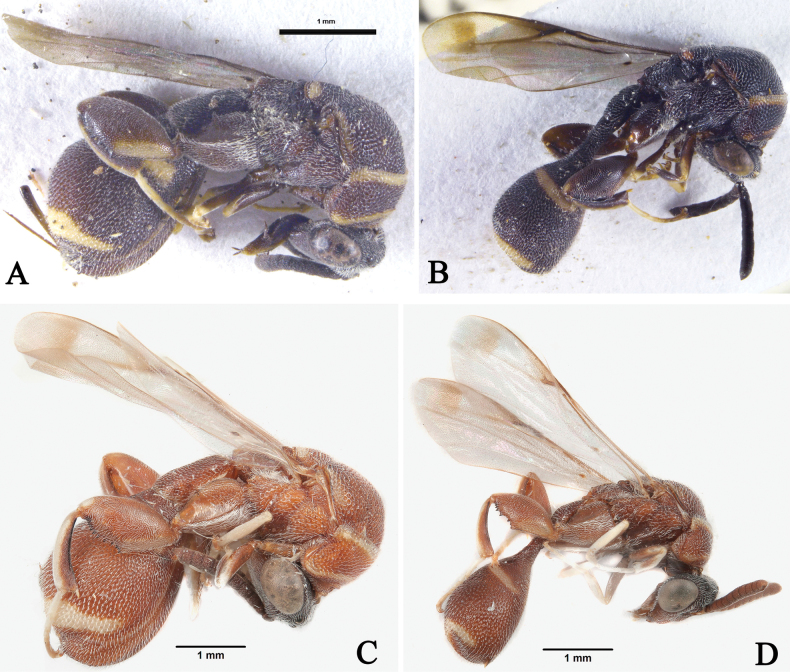
*Micrapionclavaforme* Steffan, habitus, lateral view **A, B** dark brown-pale yellow morph **A** female **B** male **C, D** reddish brown-yellow morph **C** female **D** male.

###### Remarks.

Females and males were collected in the present study from two sites in Saudi Arabia. They agree fairly well with the original description of *M.clavaforme* and the diagnoses by Bouček (1974) and Gadallah et al. (2018). In both sexes there are two colour morphs, one brown with ivory stripes and another reddish brown with yellow stripes. All specimens, however, exhibit almost no variation in stripe patterns and wing infuscation.

Schmid-Egger (2010) tentatively identified two *Leucospis* specimens from Yemen as L.aff.namibica. On close examination of his figure (Schmid-Egger 2010: 321, pl. 3) there is no doubt that these specimens are not a *Leucospis* species but *Micrapion* Kriechbaumer instead. Here, these specimens are re-identified as *M.clavaforme*.

###### Host.

Solitary bees: *Ceratina* Latreille (Bouček 1974).

###### Distribution.

Afrotropical: (Steffan 1948; Bouček 1974). Saudi Arabia (Al Bahah, Asir, Najran) (Gadallah et al. 2018); Yemen (Schmid-Egger 2010 as Leucospisaff.namibica).

## Supplementary Material

XML Treatment for Leucospis ayezae

XML Treatment for Leucospis arabica

XML Treatment for Leucospis elegans

XML Treatment for Leucospis africana

XML Treatment for Leucospis insularis

XML Treatment for Micrapion clavaforme

## ﻿Additional information

### Author ORCIDs

Syed Kamran Ahmad https://orcid.org/0000-0002-6211-2345

Syeda Uzma Usman https://orcid.org/0000-0003-2578-2515

Farmanur Rahman Khan https://orcid.org/0000-0001-8906-709X

Hossein Lotfalizadeh https://orcid.org/0000-0002-7927-819X

Hassan A. Dawah https://orcid.org/0000-0001-5642-7247

Parvez Qamar Rizvi https://orcid.org/0000-0002-2965-1806

Prince Tarique Anwar https://orcid.org/0000-0002-1682-0033

### Data availability

All of the data that support the findings of this study are available in the main text.
